# Reliable electrocortical dynamics of target-directed pass-kicks

**DOI:** 10.1007/s11571-024-10094-0

**Published:** 2024-03-16

**Authors:** Daghan Piskin, Daniel Büchel, Tim Lehmann, Jochen Baumeister

**Affiliations:** Exercise Science and Neuroscience Unit, Department Sport and Health, Paderborn University, Warburger Straße 100, 33100 Paderborn, Germany

**Keywords:** EEG, Electrocortical, Accuracy, Soccer, Kicking, Reliability

## Abstract

Football is one of the most played sports in the world and kicking with adequate accuracy increases the likelihood of winning a competition. Although studies with different target-directed movements underline the role of distinctive cortical activity on superior accuracy, little is known about cortical dynamics associated with kicking. Mobile electroencephalography is a popular tool to investigate cortical modulations during movement, however, inherent and artefact-related pitfalls may obscure the reliability of functional sources and their activity. The purpose of this study was therefore to describe consistent cortical dynamics underlying target-directed pass-kicks based on test–retest reliability estimates. Eleven participants performed a target-directed kicking task at two different sessions within one week. Electroencephalography was recorded using a 65-channel mobile system and behavioural data were collected including motion range, acceleration and accuracy performance. Functional sources were identified using independent component analysis and clustered in two steps with the components of first and subsequently both sessions. Reliability estimates of event-related spectral perturbations were computed pixel-wise for participants contributing with components of both sessions. The parieto-occipital and frontal clusters were reproducible for the same majority of the sample at both sessions. Their activity showed consistent alpha desyhronization and theta sychnronisation patterns with substantial reliability estimates revealing visual and attentional demands in different phases of kicking. The findings of our study reveal prominent cortical demands during the execution of a target-directed kick which may be considered in practical implementations and provide promising academic prospects in the comprehension and investigation of cortical activity associated with target-directed movements.

**Supplementary Information:**

The online version contains supplementary material available at 10.1007/s11571-024-10094-0.

## Introduction

Target-directed and accuracy-demanding movements exist in the nature of many sport types. Athletes throw, hit or pass a ball towards a certain target, such as a hoop in basketball, a cup in golf or teammates as in rugby. Hitting this target accurately and on time increases the likelihood of scoring, and thus winning a competition (Lyons et al. 2006; van den Tillaar and Fuglstad 2017). For football, one of the most played sports in the world, kicks are a “sine qua non” action used by players either to pass the ball from one position to another, or to perform a goal (Andersen and Dorge 2011; Kunz 2007). Kicking accuracy alone or accompanied by kicking speed is an indicator of a good manoeuvre in terms of passing the ball to the correct spot at the correct time (Bauer 1993; Hunter et al. 2018). A recent review has described accuracy as one of the most significant variables for determining success in football (Lepschy et al. 2018). An accurate kick requires coding, storing and translating spatial information into an appropriate motor output, making it a complex target-directed movement with high cortical demands (Davids et al. 2000). However, cortical dynamics associated with target-directed kicking has been barely investigated, probably due to methodological challenges in capturing cortical activity during movement. Only two recent mobile brain imaging studies have focused on cortical modulations which may correlate with measures of kicking performance, such as accuracy and ball velocity. Palucci-Vieira et al. (2022) have revealed the impact of frontal and occipital areas on ball velocity and radial error, respectively, whereas the findings of Slutter et al. (2021) showed that higher oxygenation of the prefrontal cortex may lead to poorer accuracy while performing penalty kicks. Similarly, other EEG studies highlighted distinctive modulations in the frontal, central and posterior cortices during the superior execution of different target-directed sport movements such as golf putts, basketball free throws and archery shoots, which may propose their contribution to enhanced accuracy (Baumeister et al. 2008; Chuang et al. 2013; Rampp et al. 2022). Considering the potential role of cortical activity in movement precision, the acquisition of substantial evidence regarding cortical dynamics associated with target-directed kicking may constitute an insight for interventions which intend to improve accuracy in relevant applied scenarios, like penalty and short passes in football. It may also provide a baseline for prospective studies and help to investigate if and/or how these dynamics are affected in various contexts such as different levels of expertise, injury and interventions.

Mobile EEG is an important method to capture cortical activity during motion and in real-world environments (Palucci-Vieira et al. 2022; Park et al. 2018; Rampp et al. 2022). With its high temporal resolution, it allows to investigate movement-related cortical dynamics in healthy and clinical populations (Jungnickel et al. 2018). Time-locked analytical approaches, such as event-related spectral perturbations (ERSP), may reveal peri-movement cortical strategies used to optimise motor performance (Schranz et al. Schranz et al. 2022; Posti et al. 2021). One big challenge in EEG research with gross and complex tasks is the predisposition towards movement-related artefacts (Gorjan 2022). Further, inherent EEG problems like volume conduction results in summed projection of cortical activity onto scalp electrodes and therefore reduces spatial accuracy (Bell and Sejnowski 1995). In this regard, independent component analysis (ICA) is one of the milestone algorithms commonly used in mobile EEG studies allowing decomposition of the sum signal into mathematically independent brain and non-brain sources (Jung et al. 2000). Compared to channel-based designation of regions of interest, source-based approaches may reveal cortical sources in a more sterile manner overcoming the spatial mixing fact and become the gold standard in mobile EEG investigations (Gwin et al. 2010; Peterson and Ferris 2018; Visser et al. 2022). However, the reliability of IC-derived clusters in gross motor tasks remains inconclusive due to artifact predisposition and aforementioned pitfalls. High intra-individual variability across sessions may hinder the identification of consistent task-related markers and the reliable use of EEG especially in longitudinal studies (Mayeux 2004). Aware of this problem, several EEG studies have assessed the test–retest reliability of diverse EEG measures in exercise and movement context with a repeated-measures design (Büchel et al. 2021; Domingos et al. 2023; Espenhahn et al. 2017). High test–retest reliability profile for a given EEG measure stands for low within-individual variance and may therefore indicate task-related states with high internal consistency (Lopez et al. 2023). For source-based analysis, only one study (Grandchamp et al. 2012) has focused on the reproducibility of ICs up to now, however, in a static task and on a correlational basis. Despite its potential advantages, the unknown test–retest reliability of functional IC activity in a gross motor task such as kicking leaves the use of source-based analysis in describing reliable task-related patterns questionable.

Based on this background, the aim of this study was i) to describe cortical dynamics associated with target-directed kicking based on source-based analysis and ii) to assess the test–retest reliability of these dynamics across two sessions within one week in young healthy novices. The primary advantage of this design is that it can reproduce cortical dynamics across different experimental sessions and reveal consistent patterns associated with kicking which do not fluctuate over time. Furthermore, functional IC dynamics with high internal consistency may provide a reliable measure for studies focusing on intra-individual changes in different scenarios such as injury and fatigue, or longitudinal changes such as response to specific interventions.

## Methods

### Participants

Eleven healthy participants (3 female/8 male, mean age: 27.42 ± 3.68), who were not engaged in sports on a regular basis, participated in this study. Only right-dominant participants were recruited to avoid hemispheric bias (Marcori et al. 2020). The laterality of the participants was determined based on Lateral Preference Inventory (Coren and Porac 1978). None of them had previous orthopaedic injuries or neurological diseases. Their activity level was assessed with the Marx Activity Scale (Marx et al. 2001) and an additional questionnaire, which indicated activity only at a recreational level. All subjects had normal or corrected vision at the time of the experiment. They were informed about the purpose and procedures of the study and gave written consent prior to participation. The ethical committee of the affiliated university approved the conduction of this study in accordance with the Declaration of Helsinki.

### Target-directed kicking task

**Fig. 1:**
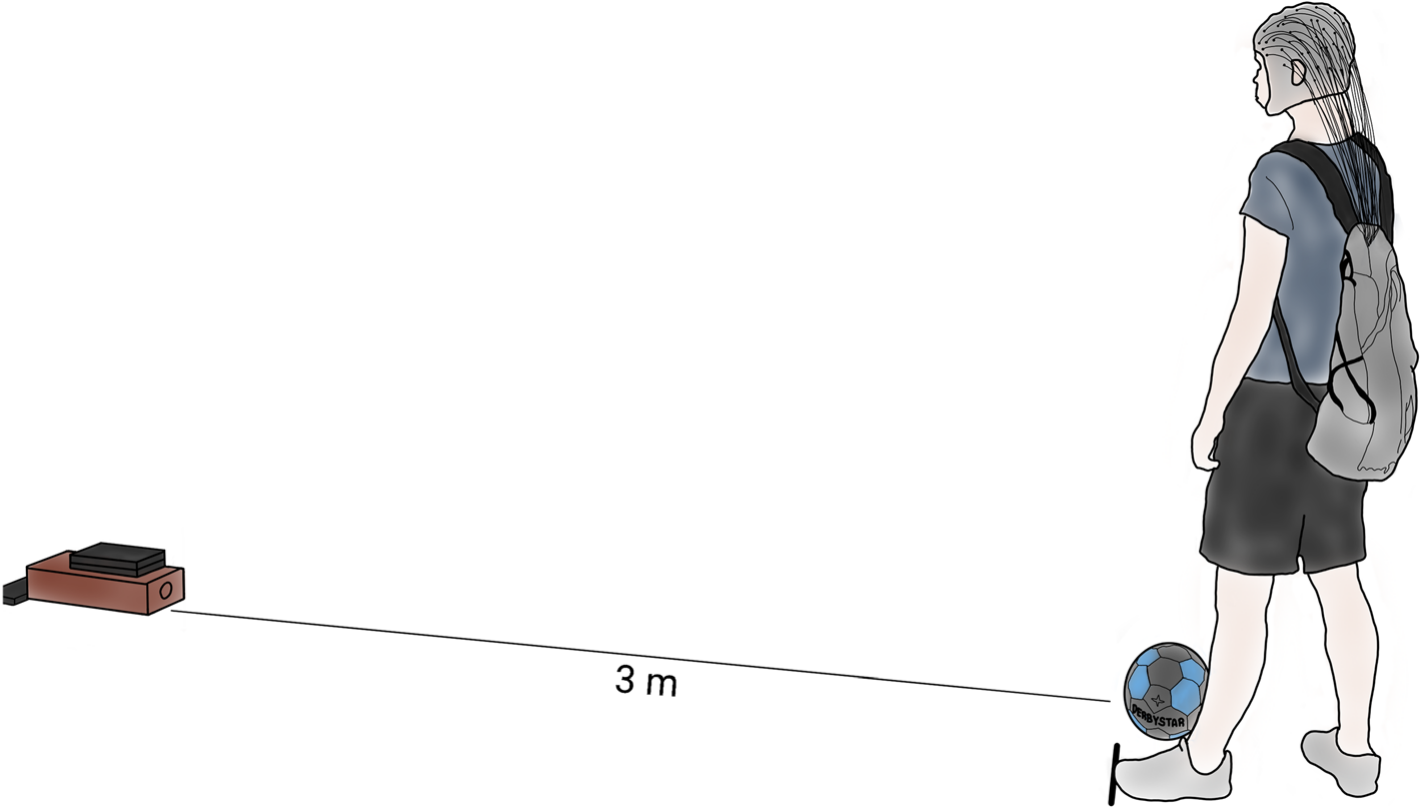
The experimental setting. Participants performed pass-kicks towards a target placed at three meters with an EEG cap, an amplifier placed in a backbag and IMU sensors attached on the legs and lower back

The present study adopted a simplified kicking task in order to ensure a stable, comparable movement pattern among novices. The target was the rectangular frontal surface (10 × 15 cm) of a wooden box fixated on the floor. In contrast to larger goals on the field, a much smaller and precise target was chosen in order to eliminate the vertical dimension of accuracy and have a concrete description of precision (Hunter et al. 2018). A standard soccer ball (FIFA size 5) was placed three metres apart from and perpendicular to the target. Participants placed their left foot next to and their right foot behind the ball with a light external rotation and were instructed to perform a pass-kick with the inside of their foot (Zago et al. 2014). They performed 10 trial-kicks to familiarise with the task and during these kicks, they were allowed to fine-tune the position of their feet to assign the most comfortable location, which was subsequently marked on the floor for the stance leg to assure a standard position for all trials. Finally, in this position, self-initiated pass-kicks were performed with the dominant (right) leg towards the target as accurately as possible. The trials in which the participants were able to hit the rectangular surface with the ball were counted as hits. In total, 90 trials were performed within six blocks of 15 trials. An overview of the experimental setting is presented in Fig. 1.

### Behavioural data collection and analysis

The three dimensional biomechanics of the kicking and stance leg were recorded using a wearable inertial measurement unit (IMU) system (myoMOTION, Noraxon, USA) at a sampling rate of 200 Hz. Seven IMU sensors were placed on the pelvis (sacral surface), thighs (laterally on the lower quadrant), shanks (anteromedially on tibial surface) and feet (on metatarsal surface dorsally and proximally to the ankle; Berner et al. 2020). The recorded biomechanical data was digitised and exported using myoRESEARCH Software (version 3.14, Noraxon, USA). The maximum acceleration of the kicking foot in the x-axis corresponding to ball contact (Lees et al. 2010) was extracted for each kick using MATLAB (version R2020b, The Math Works, USA). The minimum, maximum and median values among all 90 kicks were calculated for each participant. To ensure that participants performed pass-kicks with comparable biomechanics described in other studies, the movement range of hip flexion, knee flexion and foot external rotation was computed (Kellis and Katis 2007; Zago et al. 2014). Additionally, the accuracy performance was video-recorded using a webcam (Logitech Brio, Switzerland) synchronised with the aforementioned software. The number of hits was determined and the accuracy rate was calculated in percentage as the proportion of hits to the total number of kicks (Lepschy et al. 2018). In order to verify a comparable kicking pattern, speed and accuracy performance between two sessions, the test–retest reliability of the movement range for the aforementioned joints, minimum, maximum and median acceleration values of the kicking foot and accuracy rate were computed based on intraclass correlation coefficients (ICC; Henriksen et al. 2004).

### EEG data collection and analysis

Cortical activity was continuously recorded throughout the experiment using 65 active electrodes (actiCap, Brain Products, Germany) and a mobile amplifier (LiveAmp64, Brain Products, Germany). The electrodes were positioned in accordance with the international 10–20 system, with AFz being the ground and FCz being the reference electrode (Pivik et al. 1993). A 3D acceleration sensor (Brain Products, Germany) directly connected to the mobile amplifier was placed on the posterior of the lateral malleolus to detect onset of the kicks. The impedance was reduced to 25 kΩ and the EEG signal was recorded using BrainVision Recorder (Brain Products, Germany) at a sampling rate of 500 Hz.

The recorded EEG data were processed using MATLAB (version R2020b, The Math Works, USA) and the EEGLAB toolbox (version 14.1.2b, Delorme and Makeig 2004). Sinusoidal line noise was removed using the Cleanline plugin (Mullen 2012) and the signal was band-pass filtered between 3 and 30 Hz. Automatic detection of noisy channels was based on a deviation criterion, and the channels, whose robust z-score of standard deviation was more than 5, were removed. To avoid bias in the repeated measures design due to the unequal number of channels per session, mssing channels were interpolated before running ICA (Bidgeley-Shamlo et al. 2015). The data were then re-referenced to a common average and downsampled to 256 Hz.

The preprocessed data were epoched based on kick-onset. The kick-onset was detected based on the 3D acceleration data as the point where a statistically significant increase was observed in the transverse plane, namely the x-axis (Lees et al. 2010). The acceleration signal was rectified and smoothed with a Gaussian-weighted moving average filter at a window length of 1000 (Mendi et al. 2013). Subsequently, abrupt changes in the resulting signal corresponding to kick-onset were determined on the basis of signal mean using the *ischange* function in Matlab. In order to focus on peri-event cortical modulations such as movement planning, execution and monitoring, an epoch time window of 3000 ms before and after kick-onset was chosen with the following reasons: (i) Preparatory cortical processes have been shown to emerge two seconds before the movement onset (Shibasaki and Hallett 2006), (ii) The approximate duration of the kicks in our data was two seconds (0–1000 ms: backswing and swing, 1000–2000 ms: follow-through), (iii) Shortening of epochs after wavelet transformation. Upon the visual inspection of epochs, those containing non-stereotypical artefacts were rejected. Baseline correction was performed from − 2500 to − 2000 ms (Groppe et al. 2009) and the data were decomposed into maximally independent sources of cortical activity using an adaptive mixture independent component analysis (AMICA; Palmer et al. 2012). To avoid the effect of rank deficiency due to spherical interpolation, the dimension of the analysis of the principal components was reduced relative to the number of channels interpolated (Bidgeley-Shamlo et al. 2015). The spatial source of decomposed independent components (IC) was estimated using a standardised four-shell spherical head model (BESA, Germany) implemented in the DIPFIT plugin (Oostenveld and Oostendorp 2002). Correspondingly, brain components were labelled based on their source, activity and residual variance (≤ 15%, Onton and Makeig 2006). The brain components detected in the first session and subsequently in both sessions were clustered using the *k*-means algorithm in two different steps. To avoid circular inference in the subsequent statistical analysis, clustering was only based on dipole locations (Kriegeskorte et al. 2009). ICs located more than three SDs apart from cluster centroids were set as outliers. The number of clusters was specified using the Silhouette, Davies-Bouldin and Calinski-Harabasz optimisation algorithms based on the mean distance of ICs to cluster centroids (Miyakoshi et al. 2020). Reproducible clusters representing at least 50% of the sample in both clustering steps and including ICs of both sessions in at least 50% of the sample in the second clustering step (Peterson and Ferris 2018; Solis-Escalante et al. 2019) were assumed to be prominent cortical areas involved in kicking and considered in reliability analysis.

### Computing ICC estimates based on ERSPs

For the reproduced clusters, participants contributing with ICs of both sessions were retained. For each participant and IC, the ERSP matrix was extracted for session I and II and individually for the frequency ranges of interest, namely theta (4–7 Hz), alpha-1 (8–10 Hz), alpha-2 (11–13 Hz) and beta-1 (14–20 Hz) using the integrated study function in EEGLAB. Due to possible contamination caused by muscular activity at frequencies higher than 20 Hz, the beta-2 range was sidelined (Paluch et al. 2017). The corresponding ERSP matrices were accessed in MATLAB and for participants with multiple ICs in a session, the ERSP values were averaged on a pixel basis in order to transform multiple ICs into one representative array for a single session (Delorme and Makeig 2004). This procedure ended up in a 50 × 200 matrix with each cell representing a single pixel in the ERSP map with *x* axis corresponding to epoch time points and *y* axis to frequency. Subsequently, the *ICC* estimates (*r* values) and the corresponding *p*-values were computed for each pixel in order to investigate test–retest reliability (Shrout and Fleiss 1979). With this approach, a resultant reliability map was achieved at which the *x-* and *y*-axes indicated time and frequency, respectively, and the consistency in ERSP patterns between session I and I was demonstrated (the visualisation of ICC estimation protocol is presented in online source 1). Finally, for the interpretation of cortical dynamics in a systematic manner, the *r* values were averaged for four frequency bands of interest (theta, alpha-1, alpha-2 and beta-1) and for each 250 ms in a 4 × 20 matrix.

### Statistical analysis

All statistical analyses were performed in MATLAB (Version 2020b, The Math Works, USA). Initially, the normality of the acceleration data was assessed using MATLAB’s *swft* function (Gardner-O’Kearny 2021) and the median value was adopted to indicate the average peak acceleration for each participant due to skewed distribution. The relative test–retest reliability of behavioural and cortical data was investigated using *ICC* function (Salarian 2021). The *ICC* estimates and their 95% confidence intervals (*CI*) were computed with the following formula of a two-way mixed effects model, based on single ratings and absolute agreement (McGraw and Wong 1996; MSBS = mean square between subjects; MSE = mean square for error; *k* = number of measurements; *n* = number of subjects; MSBM = mean square between measurements).

$$ICC = \frac{MSBS - MSE}{{MSBS + \left( {k - 1} \right)MSE + \frac{k}{n}\left( {MSBM - MSE} \right) }}$$

An *ICC* value is conventionally a positive value between 0 and 1 (negative estimates in some cases), with values between 0.5 and 0.75 indicating moderate reliability, 0.75 and 0.9 good reliability and > 0.9 excellent reliability (Koo and Li 2016). In the current study, these values were adopted to rate reliability and the negative *ICC* scores, as well as the pixels with a non-significant *p*-value, were treated as zeros (Zhang et al. 2011; Zhang et al. 2017). Systematic differences resulting from random variation between two time points were inspected using paired *t*-test.

## Results

### Behavioural data

**Fig. 2:**
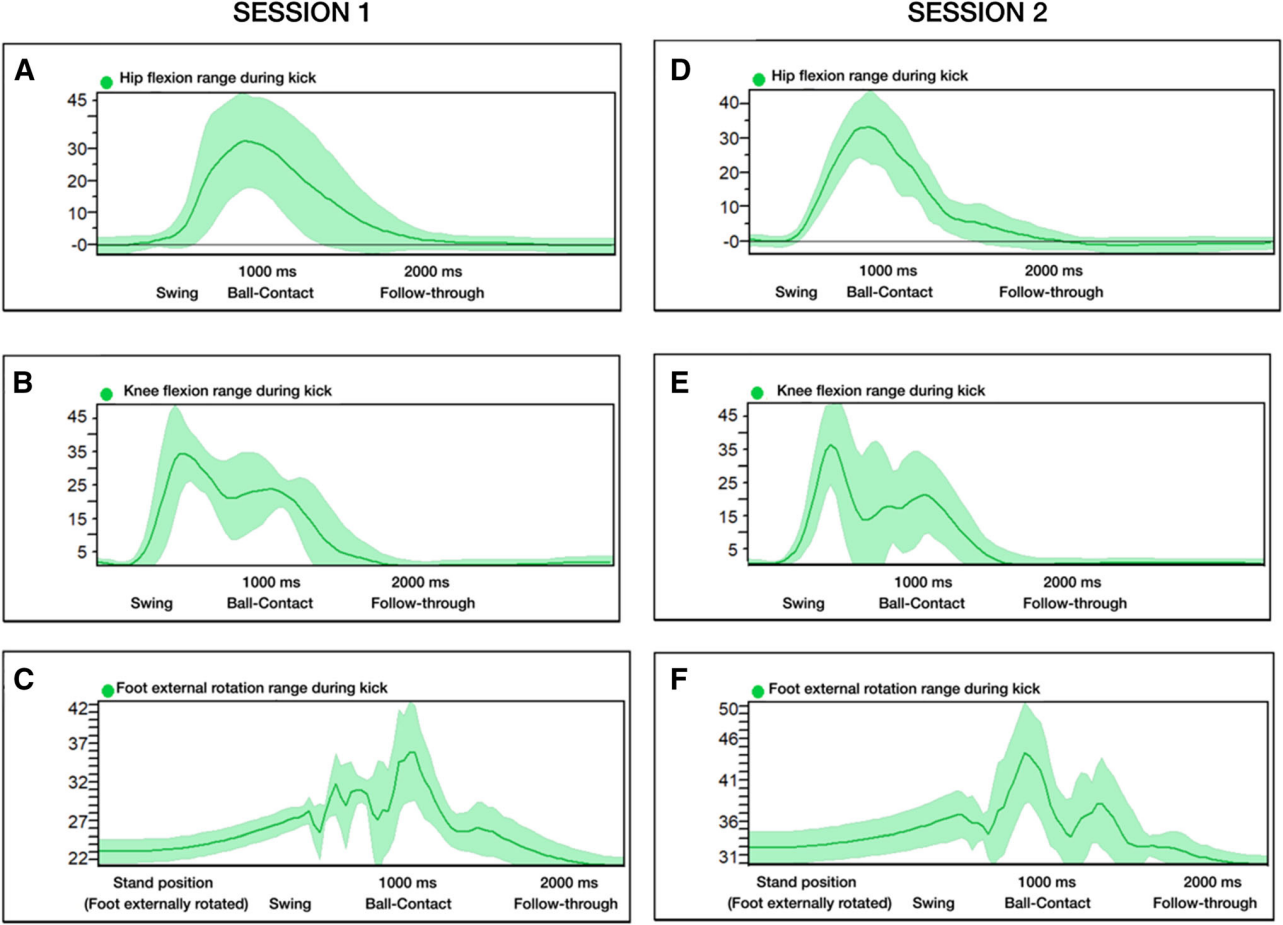
Movement range of hip flexion, knee flexion and foot rotation during kicking in session I (**A**, **B** and **C**) and session II (**D**, **E** and **F**). The comparable behavioural pattern in swing and follow-through phases yielded moderate reliability for each of the described anatomical motions

The analysis of the biomechanical data demonstrated that participants performed pass-kicks with backswing and swing phases at both sessions. The hip flexion, knee flexion and foot external rotation components are shown in Fig. 2. The movement range of hip flexion, knee flexion and foot external rotation showed moderate reliability from session I to II (*ICC* = 0.65, *CI* = 0.02–0.92; *ICC* = 0.62, *CI* = 0.06–0.89; *ICC* = 0.74, *CI* = 0.20–0.94 respectively). There were no systematic differences in any of the mentioned movement ranges between both sessions.

The minimum and the median values of the peak acceleration showed moderate reliability (*ICC* = 0.68, *CI* = 0.16–0.91; *ICC* = 0.57, *CI* =  − 0.02 to 0.87 respectively), whereas the maximum value showed poor reliability (*ICC* = 0.25, *CI* =  − 0.39 to 0.74). No systematic differences were found between sessions.

The accuracy rate showed moderate reliability (*ICC* = 0.56, *CI* =  − 0.002 to 0.86) from session I (*M* = 63.94, *SD* = 12.04) to II (*M* = 66.19, *SD* = 12.45, for indivudual accuracy rates please see online source 2).

### Cortical clusters

**Fig. 3:**
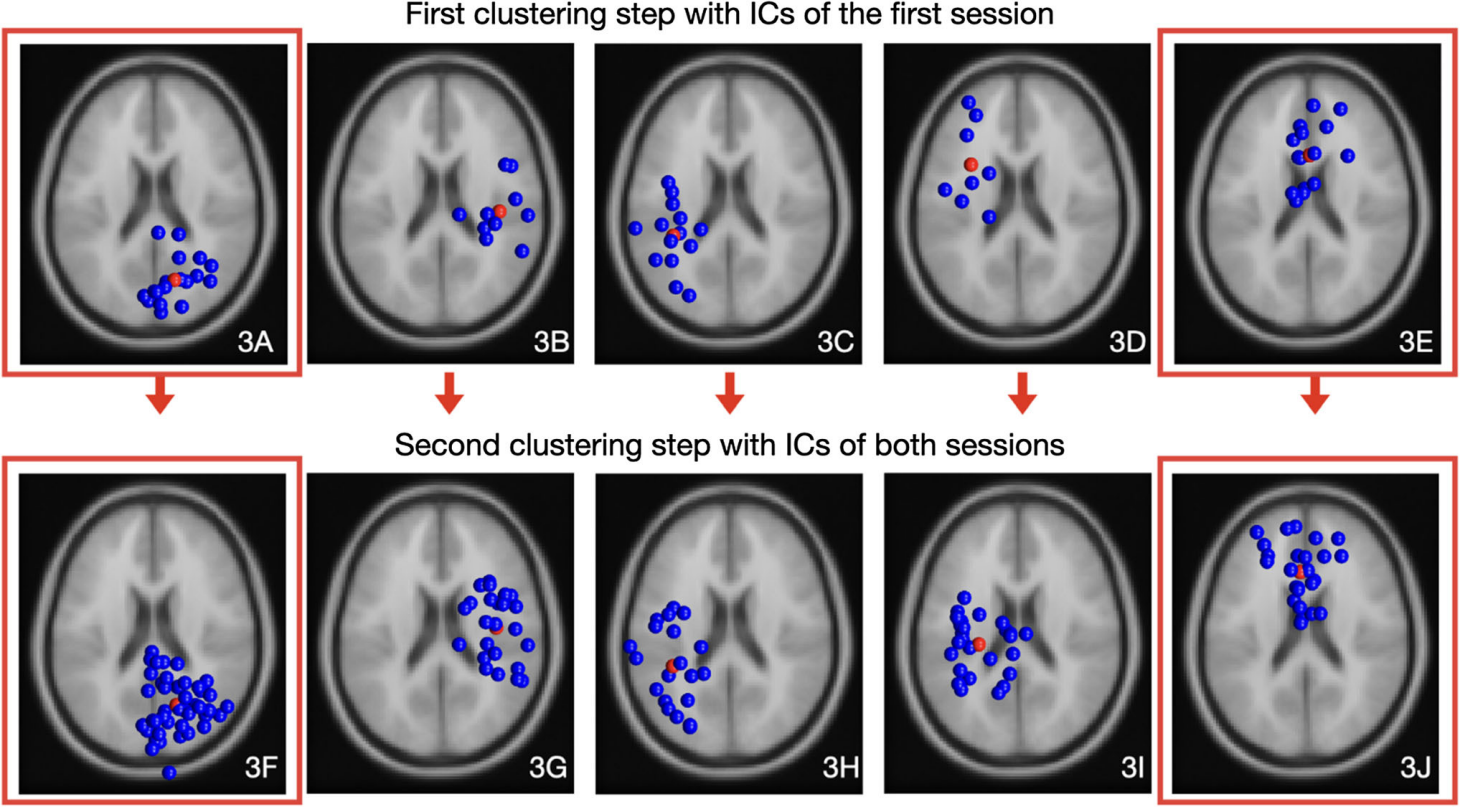
Obtained clusters in the first (**A**–**E**) and second clustering step (**F**–**J**) including ICs of only the first and subsequently both sessions. The parieto-occipital and frontal clusters (**A** and **F**; **E** and **J**; marked in red) were reproducible at the second session and considered for reliability analysis. They included independent components of the first session for 100% and 73% of the sample in the first clustering step and components of both sessions for the 100% and 64% of the sample in the second clustering step, respectively. (Color figure online)

**Fig. 4:**
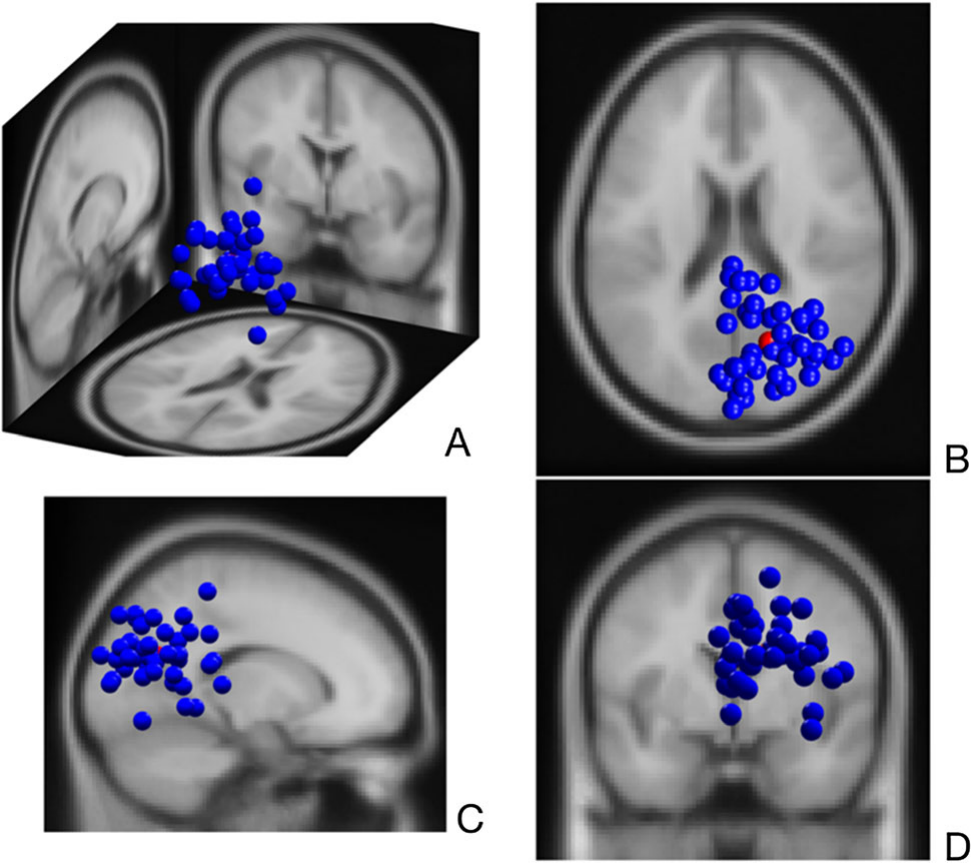
Dipoles of the right parieto-occipital cluster (11 participants, 43 ICs) with a 3D (**A**), top (**B**), sagittal (**C**) and coronal (**D**) view

**Fig. 5:**
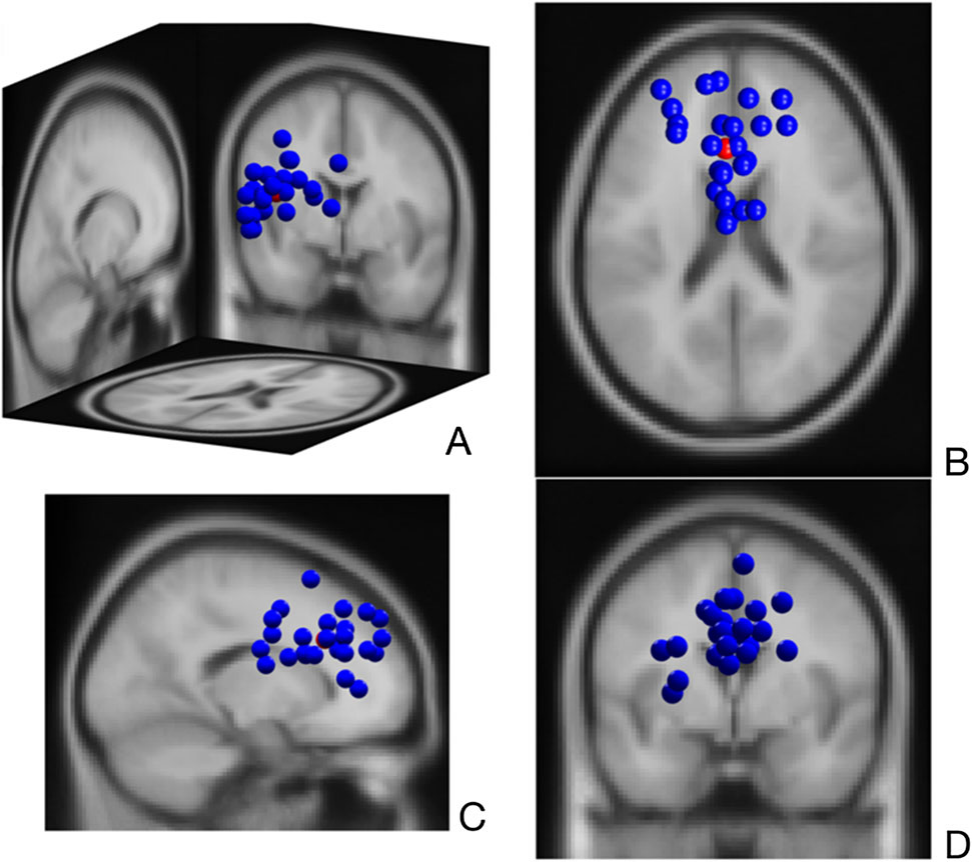
Dipoles of the mid-frontal cluster (7 participants, 25 ICs) with a 3D (**A**), top (**B**), sagittal (**C**) and coronal (**D**) view

The optimum number of clusters suggested by the three optimisation algorithms (Silhouette, Davies-Bouldin and Calinski-Harabasz) was five for both clustering steps. The cortical data recorded at the first session revealed a right parieto-occipital cluster (11 participants, 18 ICs, Fig. 3A), a right fronto-parietal cluster (5 participants, 10 ICs, Fig. 3B), a left parieto-occipital cluster (6 participants, 14 ICs, Fig. 3C), a left frontal cluster (5 participants, 8 ICs, Fig. 3D) and a mid-frontal cluster (8 participants, 15 ICs, Fig. 3E). The second clustering step with ICs of both sessions revealed a right parieto-occipital cluster (11 participants, 43 ICs, Fig. 3F), a right fronto-parietal cluster (10 participants ICs, 24 ICs, Fig. 3G), a left parieto-occipital cluster (6 participants, 20 ICs, Fig. 3H), a left fronto-parietal cluster (10 participants, 27 ICs, Fig. 3I) and a mid-frontal cluster (7 participants, 25 ICs, Fig. 3J). The number of components per participant showed moderate to excellent reliability from session one to two (*ICC* = 0.87, CI = 0.61–0.96). The right parieto-occipital (Fig. 4) and the mid-frontal cluster (Fig. 5) represented at least 50% of the participants at both time points and included components from both sessions for at least 50% of the participants (100% and 64% respectively), and were considered for reliability analysis.

### ERSPs and their reliability map

**Fig. 6:**
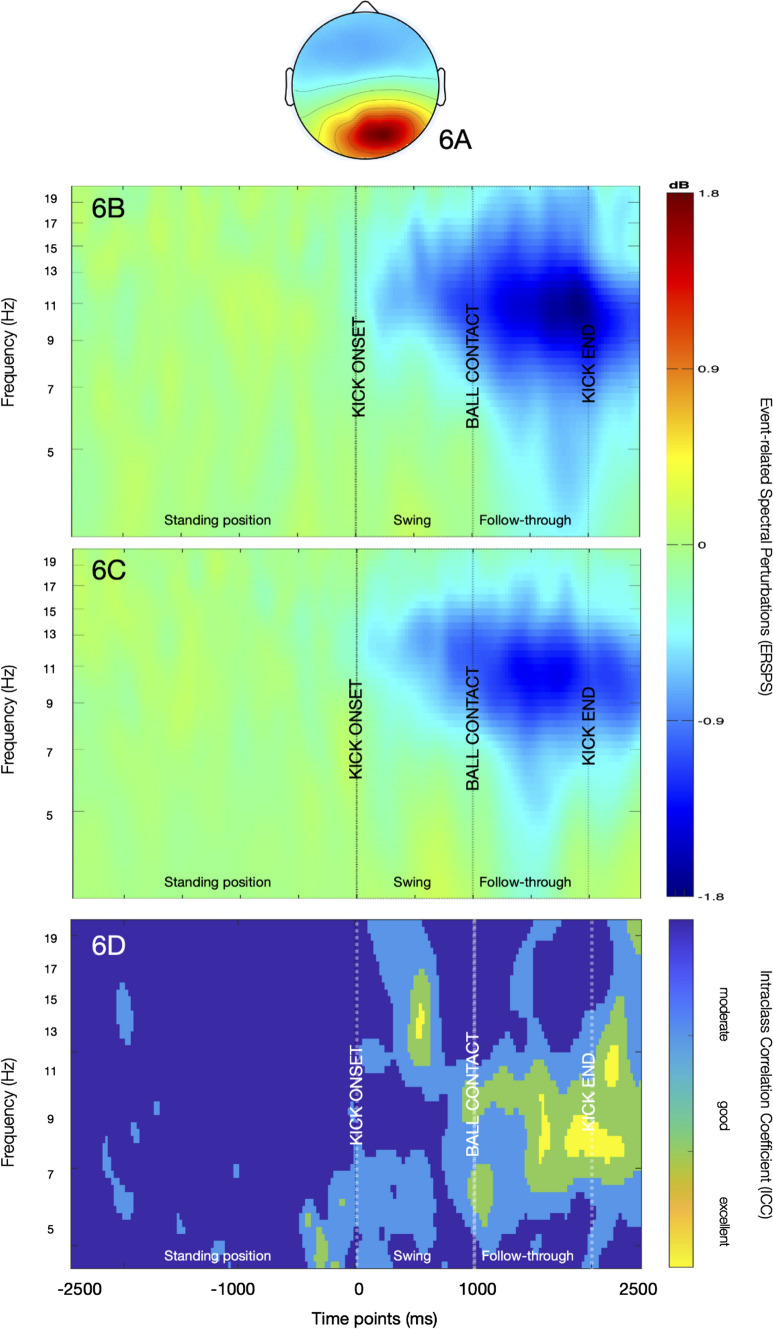
The scalp map (**A**) and ERSPs of the parieto-occipital cluster for the first (**B**) and second (**C**) session. An alpha desynchronisation (blue pattern) can be observed emerging in the swing phase and becoming stronger in the follow-through phase. The reliability map (**D**) shows moderate to excellent *ICC*s for this desynchronisation with higher scores towards kick-end. (Color figure online)

**Fig. 7:**
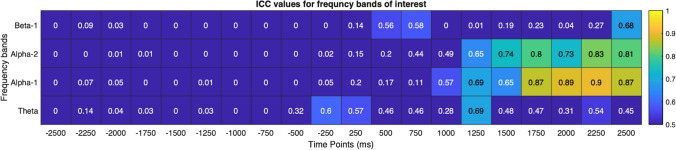
Averaged *ICC* estimates of the parieto-occipital cluster for theta, alpha-1, alpha-2 and beta-1 frequency ranges indicating higher reliability following kick onset (0–2500 ms). The estimates are higher especially for Alpha-1 range

ERSPs of the right parieto-occipital cluster (Fig. 6) demonstrated a strong desynchronization in the alpha band upon kick-onset at both sessions. The desynchronization emerged in the alpha-2 band in the swing phase, radiated to alpha-1 and slightly to beta-1 bands following ball contact, becoming more evident prior to ball contact between 1000 and 2000 ms in the alpha-2 band. The *ICC* estimates showed moderate to excellent reliability for these frequency bands after kick-onset with higher scores after 1000 ms overlapping with the stronger desynchronisation in the follow-through phase. The averaged *ICC* estimates are presented in Fig. 7.

**Fig. 8:**
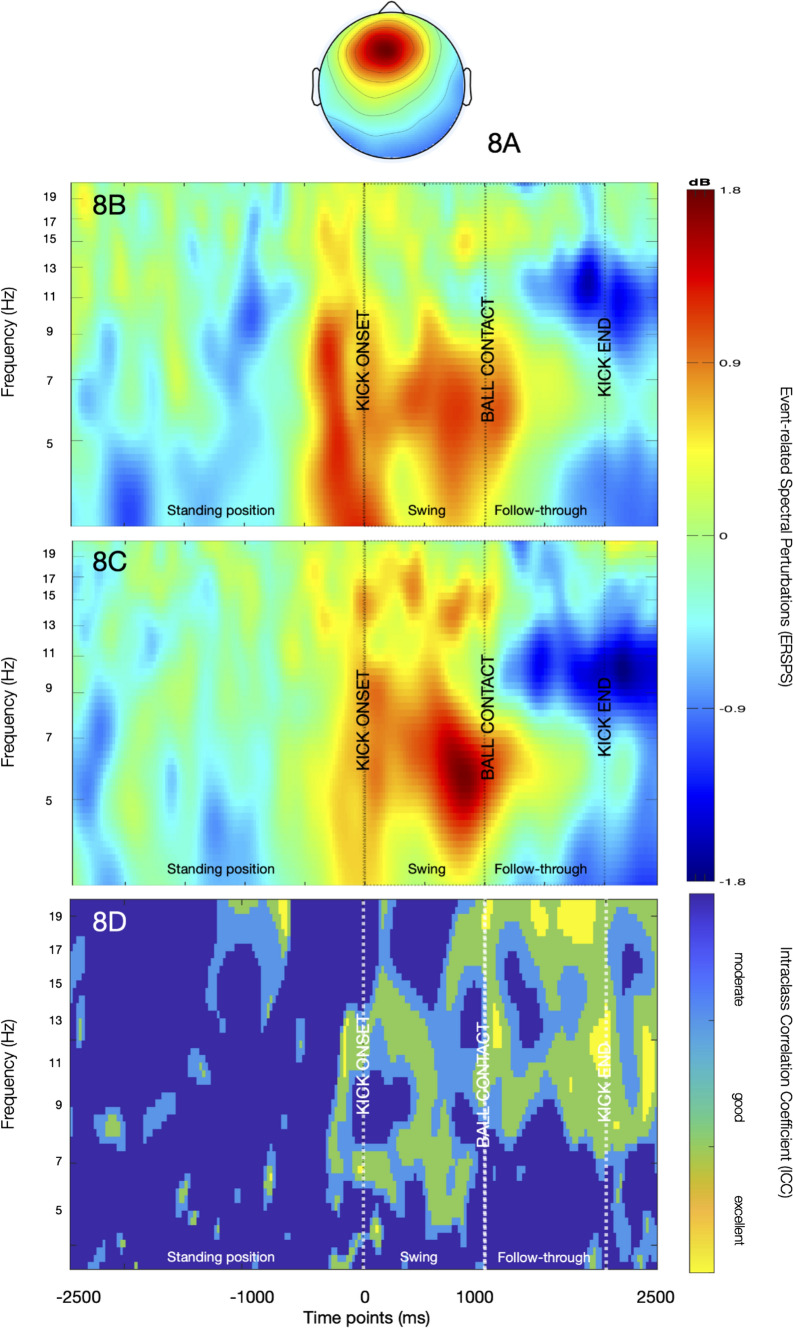
The scalp map (**A**) and ERSPs of the mid-frontal cluster for the first (**B**) and second (**C**) session. A theta synchronisation (red pattern) can be seen emerging prior to kick-onset and becoming stronger at ball contact. A subsequent desynchronisation (blue pattern) can be observed in the alpha band starting in the follow-through phase and continuing after kick-end

**Fig. 9:**
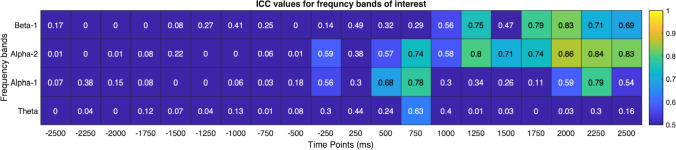
Averaged *ICC* estimates of the mid-frontal cluster for theta, alpha-1, alpha-2 and beta-1 frequency ranges indicating higher reliability following kick onset (0–2500 ms). (Color figure online)

ERSPs of the mid-frontal cluster (Fig. 8) revealed a theta synchronization starting prior to ball-contact and continuing in the swing phase. At both sessions, the synchronization was distinctive after kick-onset and in the second half of the swing phase towards ball contact. Furthermore, a desynchronization could also be seen in the alpha band starting in the follow-through phase and becoming stronger peri-kick-end. The *ICC* estimates showed moderate to good reliability for the theta synchronization observed at kick-onset and in the swing phase before ball contact until 1000 ms. For the alpha desynchronization seen after ball contact, the *ICC* estimates demonstrated moderate to excellent reliability. The averaged *ICC* estimates are presented in Fig. 9.

## Discussion

The purpose of the current study was to identify prominent cortical dynamics which contribute to target-directed pass-kicks in novices and show substantial reliability across two different sessions. The main findings reveal the reliable activity of right parieto-occipital and mid-frontal clusters observed in the same majority of the sample at both sessions. The kick-related activity of these clusters was especially evident following kick-onset and showed substantial reliability from session to session as measured by kick-related spectral perturbations. The alpha desynchronisation in the parieto-occipital ERSPs after kick-onset demonstrated moderate to excellent reliability. The reliability of the frontal theta synchronisation in the swing phase was good, whereas estimates for alpha desynchronisation showed good to excellent reliability in the follow-through and post-kick phases. Moreover, the behavioural measures—specifically anatomical motion, acceleration and accuracy rate—showed moderate reliability, supporting the assumption that the kicking behaviour associated with the observed cortical dynamics was comparable between the two sessions. These findings may suggest that the consistent kick-related activity of right parieto-occipital and frontal areas may represent reliable cortical dynamics contributing to different phases of target-directed kicking.

The novel approach in this study was to identify consistent cortical patterns associated with target-directed kicking based on the reliability estimates of kick-related perturbations in reproducible clusters. Although all clusters could be reproduced in the second session with similar dipole locations (Fig. 3), only the right parieto-occipital and mid-frontal clusters represented ICs of the same majority at both sessions. Grandchamp et al. (2012) have mentioned the reoccurance of specific ICs to be a potential cortical marker for a given task. Given that the posterior and frontal regions are linked to enhanced visual processing and top-down regulation respectively (Babiloni et al. 2006; Doppelmayr et al. 2008), the insistent occurance of posterior and frontal ICs at two different sessions may reflect the prominency of visual and attentive demands while trying to kick a ball towards a target as accurate as possible. Palucci-Vieira et al. (2022) have recently reported similar results indicating a correlation between the activity of frontal/posterior areas and radial error/variance of ball velocity during kicking. Other EEG studies investigating cortical mechanisms involved in golf and basketball tasks have also underlined the potential role of these areas in accuracy performance (Baumeister et al. 2008; Chuang et al. 2013), which may support the notion that target-directed tasks may characterize high visual and attentive demands as reflected by the associated activity of posterior and frontal cortices (Paneri and Gregoriou 2017).

At both sessions, the ERSP patterns in these two regions were visible especially following kick-onset in theta, alpha and beta bands (Figs. 6 and 8). A plausible reason for this may be the segmented and durative nature of kicking. Unlike instantaneous response-tasks with a very short duration and a small amplitude, kicking a ball towards a target requires the execution of sequential phases such as backswing, swing and ball contact (Kellis and Katis 2007). In the current analysis, the EEG data was epoched based on the onset of the backswing phase with the a priori assumption that preparatory dynamics may be observed in a time range of up to two seconds before movement onset (Shibasaki and Hallett 2006). However, it can be argued that the most decisive component of a kick is the ball contact, determining the direction and speed of the ball upon release. This may increase cortical demands shortly before ball contact and explain why the observed dynamics were seen not prior to, but during the execution of swing phase in contrast to our assumption (Toda et al. 2011; Waldert et al 2008).

The right lateralisation of ICs in the parieto-occipital cluster disclose the activity of right posterolateral regions during the execution of the task (Fig. 4). Given that the non-dominant hemisphere is postulated to dominate the control of visuospatial processes (Corballis 2003; Spagna et al. 2020), the visual demands of right-side kicks may be reflected predominently by the activity of non-dominant (right) posterolateral areas. The consistent alpha desychronization induced by kick-onset in this cluster (Fig. 6) may support this functional assumption, as it is referred to augmented visual processing in numerous studies (Erickson et al. 2019; Rajagovindan and Ding 2011; Worden et al. 2000). In various cognitive tasks, a high reliability for alpha response over posterolateral regions was reported in attribution to consistent visual fundamentals of the tasks (Vazquez-Marrufo et al. 2020; Neuper et al. 2005). Even though its reliability has not been assessed in movement context until now, our parallel findings indicating substantial reliability for alpha desychronization in the swing (0–1000 ms) and follow-through phase (1000–2000 ms) may reveal visual demands with high within-individual consistency, which may be linked with target detection and tracking ball trajectory (Gallicchio and Ring 2020). Furthermore, this desynchronisation was also noticeable in the lower beta range at both sessions, being relatively weaker at the second one. Gomez et al. (2006) have associated alpha and lower beta rhytms in the frontal, as well as in the posterior regions with enhanced preparatory processes in visuomotor tasks. Based on this, it may be speculated that the alpha and lower beta responses together may suggest a preparatory arousal starting in the swing phase. Moreover, posterior beta power is also shown to decrease synchronously with Rolandic beta rhythms to modulate motor excitability in visuomotor tasks, which may be another explanation for this alpha–beta coupling (Mäki and Ilmoniemi 2010). However, as suggested by moderate reliability estimates, the beta reponse may rather indicate dynamics which fluctuate from session to session and represent variable strategies as a result of habituation (Lopez et al. 2023).

The ERSPs of the frontal cluster reveal a theta synchronisation prior to and an alpha desynchronization folowing ball contact with high reliability estimates (Fig. 8). Frontal midline theta is attributed to executive processing—especially augmented attention—in cognitive (Doppelmayr et al. 2008; Laukka et al. 1995), as well as in motor tasks (Baumeister et al. 2008; Chuang et al. 2013). The consistent theta synchronisation observed prior to ball-contact at both sessions may underline the insistent need for increased focus before releasing the ball during a kick. At this phase, participants attempt to initiate the movement in line with the target and subsequently undergo fine motor adjustments under enhanced focus, such as inhibition of inappropriate leg position, in order to kick the ball with optimal accuracy. A similar theta response was also observed in other sport-specific tasks with a target-directed nature and similar demands (Baumeister et al. 2008; Chuang et al. 2013). Our results may contribute to the prominency of attentional processes before impact (ball contact in our study) in target-directed tasks as indexed by task-related theta activity, whose reliability has also been shown in other static tasks (McEvoy et al. 2000; Ding et al. 2022).

Besides theta reponse, a clear alpha desynchronisation was also remarkable in the frontal ERSPs starting subsequent to ball contact and preceded by a moderate synchronisation in the swing phase (Fig. 8). Task-related response in the frontal alpha band is mentioned to highlight top-down processes regulating the inhibition/activation of areas associated with motor tasks (Klimesch et al. 2006). From this perspective, the desynchronisation occurring after 1000 ms in the follow-through phase may be interpreted as the re-activation of areas which were inhibited prior to ball contact to avoid irrelevant information processing and maximize attention in the swing phase. The moderate, preceding sychronisation observed in the swing phase may reinforce this remark, as it may propose the inhibitory role of alpha activity prior to ball contact (Klimesch et al. 1999). Another attribute of alpha desynchronization is its contribution to memory processes (Erickson et al. 2019; Klimesch 1995; Wianda and Ross 2019). Considering the occurance of this pattern following ball-contact, it may also suggest the encoding and retrieval of successfull kicks in the motor memory, which may be processed by the supplementary motor area upon the visual feedback achieved from ball trajectory (Gallicchio and Ring 2020; Tanji 1994). However, although this pattern is observable at both session with high reliability estimates, the paucity of studies with comparable kicking tasks hinders the task-specific interpretation of it. Palucci-Vieira et al. (2022) have shown a similar alpha synchronisation over the frontal electrodes following ball contact and interpreted it within the neural eficiency framework. Still, the very short duration of this alpha increase due to a shorter epoch window in their results does not allow for a plausible comparison with our findings. The accumulation of more evidence regarding cortical responses before and following ball release may enable the reliable task- and phase-specific interpretation of cortical dynamics associated with target-directed tasks.

In order to control for the possible confounding effect of different kicking behaviour across sessions, the current study also assessed the reliability of kicking behaviour in terms of anatomical motion range, peak acceleration and accuracy performance. Except for the maximum value of peak acceleration observed in trials, all behavioural endpoints showed acceptable reliability, which may support the assumption that the observed cortical dynamics occurred under comparable motor behaviour. The poor reliability of the peak acceleration values may be explained by the novice profile of the cohort and the instructed emphasis on accuracy during the execution of the task. The exceptional trial of different speeds as a strategy—especially at the first session until the achievement of optimal kicking behaviour—might have caused a higher variance. The development of strategies from session to session may also explain the overall suboptimal reliability estimates and wide CI ranges in the behavioral data, as changes in kicking speed are linked with changes in motion range (Lees and Nolan 2002). Moreover, prioritising accuracy is known to minimise kicking speed in line with Fitts’ law (Standage et al. 2014; van den Tillaar and Fuglstad 2017). The higher reliability estimates of mean and median values of peak acceleration may therefore acknowledge that the participants favoured overall lower speeds to pursue maximal accuracy.

To the best of our knowledge, this is the first study to describe consistent cortical dynamics underlying a target-directed movement based on test–retest reliability estimates in a mobile EEG context. By revealing reproducible clusters for the same majority of participants at two different sessions, the current findings provide an insight into the cortical regions which may prominently be involved in the execution of a target-directed kick. The high test–retest reliability of task-related parieto-occipital and frontal activity advocate the prominence of visuospatial and attentional processes in subsequent phases of a target-directed kick which do not fluctuate significantly from session to session. The described dynamics may provide a basis for prospective studies with a longitudinal design, in which the response of these dynamics to a specific intervention can be investigated (Mayeux 2004). The low within-individual variance may also enable to explore if different applied scenarios, such as fatigue and injury, may disturb these dynamics, which may contribute to precision in an important football-specific task. Last but not the least, our study proposes the use of repeated-measures design as a merit in mobile EEG studies and provides an example in terms of adopting source-based analysis in longitudinal studies.

## Limitations

The methodological limitations of the present study should be recognised and considered while interpreting the current findings and adopting this methodology in prospective studies. Regarding the kicking task used, the execution of complex movements may vary inter- and intra-individually, and increase the variance of cortical dynamics. In the present study, this effect was minimised and controlled by means of standardised position, instructed execution and reliability measures of behavioural data. Still, a possible variance should be regarded. On the other hand, the methodology-related standardisation and simplification of complex tasks can reduce ecological validity and reflect real sports-situations only to a certain extent (Chang et al. 2022).

The present findings provide an insight about cortical dynamics associated with target-directed kicking, however, do not distinguish the specific patterns which contribute to successful kicks. Investigating the distinctive patterns in successful and unsuccessful kicks may deepen the knowledge about how these cortical dynamics are used as strategies in different populations (Palucci-Vieira et al. 2022).

With regard to the limitations of EEG, this study focused only on clusters, which were reproducible for the same majority of the sample at two different time points. Although the excluded clusters may also deliver important knowledge about task-related cortical dynamics, this is a common approach used also in other studies (Solis-Escalante et al. 2019; Peterson and Ferris 2018). As another point, the number of reliable brain components varied inter-individually, however, showed good reliability within individuals. It may be inferred that the amount of dominating, task-related cortical information as measured by EEG sources is intrinsic to individuals and is reliable under equivalent circumstances (Grandchamp et al. 2012). This notion also reinforces the presumption that clusters with the majority of participants contain the more prominent task-related cortical dynamics, since when the number of ICs was less than the number of clusters in some participants, the ICs were still assigned to the considered clusters. Finally, AMICA outputs approximate cortical sources and the location of these sources may deviate from the actual anatomical locations (Gramann et al. 2010). However, the repeated measures design in this study may suggest an acceptable reliability of the discussed spatial sources, as ICs of two different time points were assigned to the same cluster for the majority of participants (Grandchamp et al. 2012).

## Conclusion

The current study described the reliable cortical dynamics underlying target-directed kicking based on test–retest reliability estimates. The parieto-occipital and frontal clusters were reproducible for the same majority of the sample at two different sessions. Their activity with high test–retest reliability estimates revealed prominent, phase-specific visual and attentional demands while kicking a ball towards a target. Our findings provide an insight for practical implementations of kicking precision and important academic prospects in understanding cortical activity associated with target-directed movements. Future studies may consider the investigation of these dynamics in different applied scenarios.

## Supplementary Information

Below is the link to the electronic supplementary material.

Supplementary file 1 (TIFF 108939 KB)

Supplementary file 2 (DOCX 13 KB)
