# Supplementary material for: Reliable electrocortical dynamics of target-directed pass-kicks
Source: Cogn Neurodyn. 2024 Mar 16;18(5):2343–57. doi: 10.1007/s11571-024-10094-0 (PMC11564708; doi:10.1007/s11571-024-10094-0)
Supplement: Supplementary file 2 — (DOCX 13 KB) [file 11571_2024_10094_MOESM2_ESM.docx]

| Participant | Accuracy Rate (%) in Session 1 | Accuracy Rate (%) in Session 2 |
| --- | --- | --- |
| 1 | 85 | 68.13 |
| 2 | 75.55 | 81.11 |
| 3 | 41.66 | 62.22 |
| 4 | 67.77 | 57.77 |
| 5 | 63.33 | 73.33 |
| 6 | 64.44 | 76.67 |
| 7 | 56.66 | 68.88 |
| 8 | 51.11 | 37.78 |
| 9 | 71.11 | 75.56 |
| 10 | 56.66 | 54.44 |
| 11 | 70 | 72.22 |
